# Atherogenic Index of Plasma Predicts Outcomes in Acute Ischemic Stroke

**DOI:** 10.3389/fneur.2021.741754

**Published:** 2021-10-11

**Authors:** Hongbing Liu, Kai Liu, Lulu Pei, Shen Li, Jiawei Zhao, Ke Zhang, Ce Zong, Lu Zhao, Hui Fang, Jun Wu, Shilei Sun, Bo Song, Yuming Xu, Yuan Gao

**Affiliations:** Department of Neurology, The First Affiliated Hospital of Zhengzhou University, Zhengzhou, China

**Keywords:** stroke, prognosis, atherogenic index of plasma, predictive value, risk factors

## Abstract

**Aim:** The atherogenic index of plasma (AIP) was significantly related to adverse outcomes in patients with cardiovascular disease. Our aim was to investigate the association between AIP and adverse outcomes in acute ischemic stroke.

**Methods:** Patients with acute ischemic stroke (AIS) admitted between 2015 and 2018 were prospectively enrolled in this study. Functional outcomes were evaluated by the modified Rankin Scale (mRS). Poor outcomes were defined as mRS 3–6. The relationship of AIP with the risk of outcomes was analyzed by multivariate logistic regression models.

**Results:** A total of 1,463 patients with AIS within 24 h of symptom onset were enrolled. The poor outcome group had a significantly higher level of AIP [0.09 (−0.10 to 0.27) vs. 0.04 (−0.09 to 0.18), *p* < 0.001] compared with the good outcome group. Multivariable logistic regression analysis showed that higher AIP was associated with poor outcomes in all the stroke patients (OR 1.84, 95% CI, 1.23–2.53, *p* = 0.007), which was more evident in patients with large-artery atherosclerosis subtype (OR 1.90, 95% CI, 1.53–2.62, *p* = 0.002), but not in the other subtypes. Receiver operating curve (ROC) analysis revealed that the best predictive cutoff value of AIP was 0.112, with a sensitivity of 70.8% and a specificity of 59.2%, and the area under the ROC curves for AIP was 0.685.

**Conclusion:** AIP may be an important and independent predictor of the outcome of dysfunction in patients with AIS, especially the stroke subtype of large-artery atherosclerosis.

## Introduction

Stroke is the most common cause of disability and death and is a global health concern (1). Despite the significant reduction in stroke-related mortality, the prognosis for patients with acute ischemic stroke (AIS) remains unsatisfactory (2). There is increasing focus on identifying new prognostic markers to better classify patients at higher risk of poor prognosis. Studies have shown that atherosclerosis (AS) is the most common cause of AIS, and dyslipidemia is the most important risk factor of AS (3, 4). Many blood lipid parameters have been used to evaluate the risk of stroke outcomes, such as total cholesterol (TC), triglyceride (TG), low-density lipoprotein (LDL-C), high-density lipoprotein (HDL-C), non-HDL-C, and other parameters (5–8), but the predictive values of these indicators are still limited.

The atherogenic index of plasma (AIP) is calculated as log (TG/HDL) and reflects the levels of TG and HDL-C cholesterol. AIP, as a robust biomarker of dyslipidemia and AS, has been used to quantify comprehensive lipid levels (9). It is also considered a biomarker of coronary syndrome and metabolic syndrome (10, 11). Previous studies have demonstrated that it is positively correlated with cardiovascular disease risk (12). Notably, some studies demonstrate that AIP may be more closely associated with cardiovascular and cerebrovascular disease risk than other individual lipoprotein cholesterol concentrations alone (13). However, few studies have examined the relationship between AIP and functional outcomes in AIS. Therefore, larger sample size studies and prospective cohort studies are needed to evaluate the relationship.

In the study, we aimed to explore the relationship between AIP and functional outcomes in patients with AIS at 3 months.

## Patients and Methods

Patients enrolled in the study were from the database of the Henan Province Stroke Registry (14–16) at the First Affiliated Hospital of Zhengzhou University from January 2015 to December 2018. AIS was diagnosed according to criteria defined by the World Health Organization (17) based on neuroimaging results, patient history, and clinical data. All patients signed written informed consent, and this study was approved by the Ethics Committee of the First Affiliated Hospital of Zhengzhou University (14).

The exclusion criteria include the following aspects: (1) the time from onset to admission is more than 24 h; (2) age <18 years; (3) a history of cancer, hematologic disease, or immunosuppressant use; (4) patients without complete clinical data; (5) severe hepatic or renal diseases; (6) infectious or systematic inflammatory disease; and (7) major trauma, surgery, or loss to follow-up.

### Data Collection

Patients' baseline data were recorded in the form of paper case reports. Demographic characteristics include gender, age, systolic blood pressure, diastolic blood pressure, smoking (defined as continuous or cumulative smoking ≥6 months, or smoking at least 6 months every day), and drinking (defined as drinking alcohol at least 5 days a week (>30 g/day) for at least 6 months). Risk factors for stroke include history of stroke/TIA, diabetes, hypertension, atrial fibrillation, coronary heart disease (CHD), and lipid-lowering therapy. The National Institutes of Health Stroke Scale (NIHSS) was used by trained neurologists to assess the neurological impairment severity of baseline stroke within 24 h after admission.

Stroke subtypes were classified by two trained study neurologists according to the Trial of ORG 10172 in Acute Stroke Treatment (TOAST) criteria (18). Large-artery AS (LAA), small-artery occlusion (SAO), cardioembolism (CE), other determined causes (OC), and undetermined causes (UC) were included in AIS subtypes. The stroke of OC and UC types were combined as “other or unknown cause” group.

Laboratory examinations were routinely obtained within 24 h of admission, including white blood cell, glucose, estimated glomerular filtration rate, and lipid profiles. The lipid profiles included TC, TG, HDL-C, LDL-C, and LDL-C/HDL-C. The AIP was calculated as log (TG/HDL), and non-HDL-C was calculated by deducting HDL-C from TC.

### Follow-Up and Outcomes

The Modified Rankin Scale (mRS) was used to evaluate the prognosis of patients: (1) poor outcomes (mRS, 3–6); (2) death; and (3) disability (mRS, 2–6). Most of the patients were followed up. Follow-up was conducted by telephone. The telephone interviewer was trained and did not participate in the registration process.

### Statistical Analysis

Continuous variables were described as median or mean ± SD which were analyzed by the Mann–Whitney U test or independent Student's *t*-test. Categorical variables were described as proportions which were analyzed using the χ^2^-test. We assessed the association between AIP and 3-month prognosis by multivariate logistic regression analysis. AIP was divided into quartiles (Q1, < -0.10; Q2, ≥-0.10 and <0.08; Q3, ≥0.08 and <0.26; and Q4, ≥0.26). Three models were applied to correspond to different endpoint events. Furthermore, we adjusted for the variables in the baseline in the models, except for those with collinearity. Pearson correlation coefficients were used to evaluate the potential for collinearity between pairs of covariates in the baseline. The correlation coefficient >0.5 was considered as a threshold for collinearity. Receiver operating characteristic (ROC) analysis was further used to evaluate the predictive power of the AIP, TC, TG, HDL-C, LDL-C, and non-HDL-C to predict the prognosis. A two-tailed *p* <0.05 was considered significant. All statistical analyses were carried out by SPSS 24.0 software.

## Results

### Baseline Characteristics

**Figure 1:**
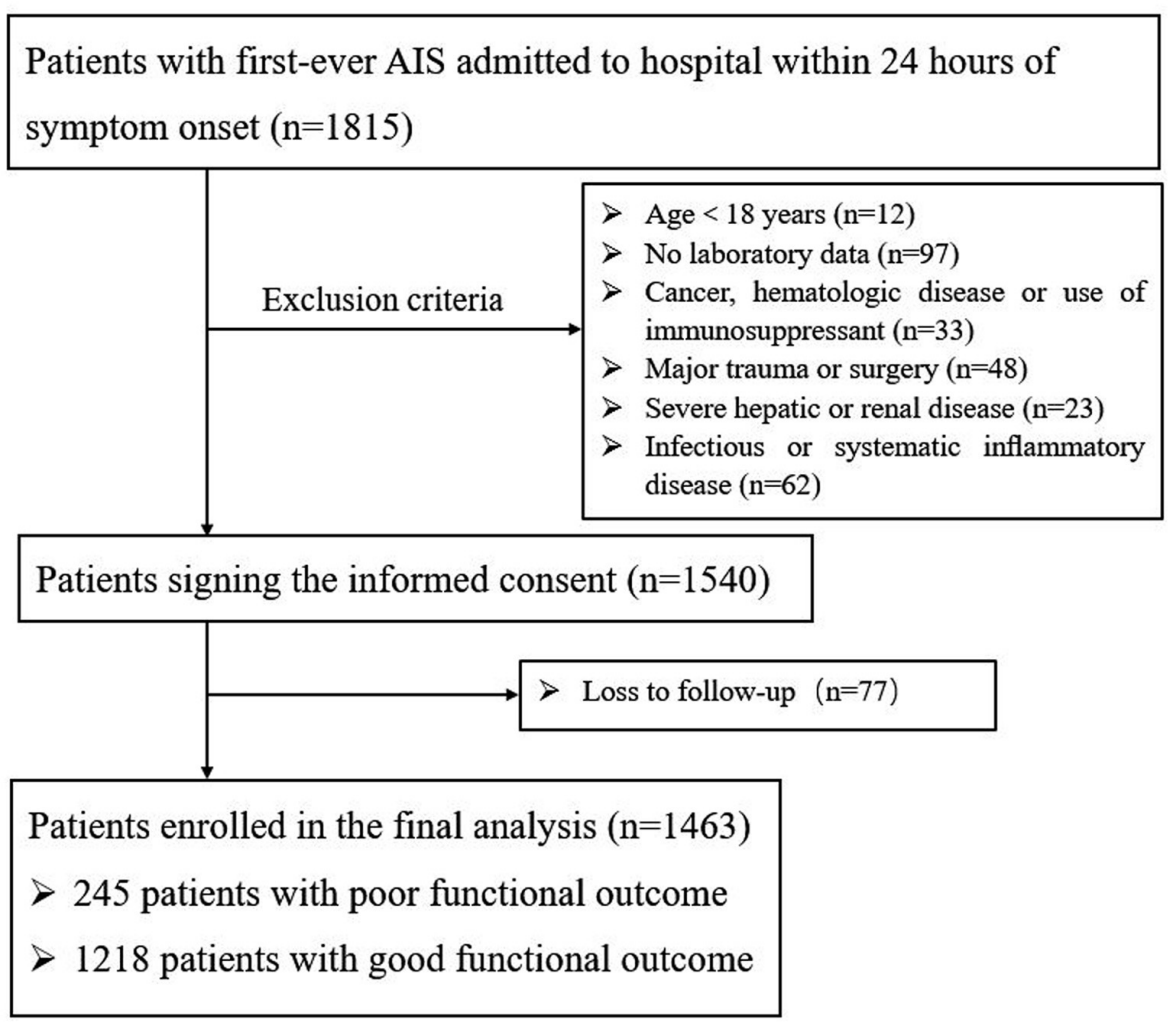
Patient flowchart of the cohort.

A total of 1,815 AIS patients were consecutively recruited within 24 h of the onset of symptoms (Figure 1). During the study period, 275 patients were excluded: 12 of the patients were under the age of 18; 97 patients had incomplete laboratory data, 118 patients had other diseases; and 48 patients underwent trauma or surgery. During 3 months of follow-up, a total of 77 patients were lost to follow-up. Finally, 1,463 patients were enrolled in the study, of whom 1,218 had a good functional outcome and 245 had a poor functional outcome. Baseline data for the two groups of patients are described in Table 1. The mean age of patients was 60.25 ± 12.31; 69.9% (1022) of them were male. As shown in Table 1 and Supplementary Figure 1 AIP [0.09 (−0.10 to 0.27) vs. 0.04 (−0.09 to 0.18), *p* < 0.001] was obviously higher than that in the poor outcome group. Compared with the good prognosis group, patients with poor prognosis were prominently older (63.86 ± 12.64 vs. 59.52 ± 12.11, *p* < 0.001), were female (35.5 vs. 29.1%, *p* = 0.045), had higher baseline NIHSS score [7 (4–12) vs. 3 (1–5), *p* < 0.001], were treated with reperfusion therapy (28.4 vs. 20.8%, *p* = 0.016), and had a history of atrial fibrillation (12.7 vs. 7.1%, *p* = 0.003) and diabetes mellitus (28.2 vs. 21.5%, *p* = 0.023). Patient laboratory parameters were also included. Glucose [5.64 (4.79–7.09) vs. 5.22 (4.48–6.93), *p* = 0.015], TG [1.15 (0.86–1.57) vs. 1.31 (0.95–1.84), *p* = 0.001], LDL-C [2.52 (1.89–3.24) vs. 2.56 (2.01–3.14), *p* = 0.011], HDL-C [1.03 (0.88–1.19) vs. 1.07 (0.91–1.28), *p* = 0.021], and non-HDL-C [3.11 (2.49–3.91) vs. 2.99 (2.37–3.61), *p* = 0.003] reached statistical significance.

**Table 1:** Characteristics of patients included according to 3-month outcomes.

**Characteristics**	**Total**	**The prognosis of 3 months**		
		**Good outcomes (*n* = 1,218)**	**Poor outcomes (*n* = 245)**	***p*-value**
Age (years)	60.25 ± 12.31	59.52 ± 12.11	63.86 ± 12.64	<0.001
Male	1,022 (69.9)	864 (70.9)	158 (64.5)	0.045
SBP (mmHg)	153.71 ± 22.82	153.22 ± 23.41	154.94 ± 21.45	0.469
DBP (mmHg)	82.12 ± 12.83	82.34 ± 13.14	81.23 ± 12.15	0.433
Smoking	679 (46.4)	571 (46.9)	108 (44.1)	0.423
Drinking	455 (31.1)	383 (31.4)	72 (29.4)	0.526
Baseline NIHSS	3 (2–6)	3 (1–5)	7 (4–12)	<0.001
Reperfusion therapy	397 (27.1)	346 (28.4)	51 (20.8)	0.016
**Medical history**				
Hypertension	871 (59.5)	715 (58.7)	156 (63.7)	0.148
CHD	181 (12.4)	147 (12.1)	34 (13.9)	0.433
Atrial fibrillation	117 (8.0)	86 (7.1)	31 (12.7)	0.003
Diabetes mellitus	331 (22.6)	262 (21.5)	69 (28.2)	0.023
Stroke/TIA	348 (23.8)	281 (23.1)	67 (27.3)	0.151
Lipid-lowering therapy	319 (21.8)	260 (21.3)	59 (24.1)	0.344
**Stroke etiology**				0.119
Large artery atherosclerosis	708 (48.4)	575 (47.2)	133 (54.3)	
Cardioembolic	168 (11.5)	149 (12.2)	19 (7.8)	
Small-vessel	313 (21.4)	262 (21.5)	51 (20.8)	
Other or unknown cause	274 (18.7)	232 (19.1)	42 (17.2)	
**Laboratory**				
WBC (10^9^/L)	7.31 (5.91–8.71)	7.22 (5.95–8.68)	7.41 (5.91–8.82)	0.113
Glucose (mmol/L)	5.36 (4.53–7.04)	5.22 (4.48–6.93)	5.64 (4.79–7.09)	0.015
eGFR (ml/min/1.73 m^2^)	92.66 (71.68–99.13)	92.99 (73.45–99.71)	88.34 (57.71–99.49)	0.228
TC (mmol/L)	4.12 (3.46–4.78)	4.11 (3.46–4.76)	4.19 (3.48–5.09)	0.077
TG (mmol/L)	1.28 (0.92–1.78)	1.31 (0.95–1.84)	1.15 (0.86–1.57)	0.001
LDL-C (mmol/L)	2.54 (2.01–3.13)	2.56 (2.01–3.14)	2.52 (1.89–3.24)	0.011
HDL-C (mmol/L)	1.06 (0.91–1.28)	1.07 (0.91–1.28)	1.03 (0.88–1.19)	0.021
Non-HDL-C (mmol/L)	2.99 (2.39–3.63)	2.99 (2.37–3.61)	3.11 (2.49–3.91)	0.003
LDL-C/HDL-C	2.39 (1.79–3.06)	2.38 (1.80–3.06)	2.51 (1.79–3.14)	0.457
AIP	0.08 (−0.09 to 0.27)	0.04 (−0.09 to 0.18)	0.09 (−0.10 to 0.27)	<0.001

*SBP, systolic blood pressure; DBP, diastolic blood pressure; NIHSS, National Institutes of Health Stroke Scale; CHD, coronary heart disease; TIA, transient ischemic attack; WBC, white blood cell; eGFR, estimated glomerular filtration rate; TC, total cholesterol; TG, triglyceride; LDL-C, low-density lipoprotein; HDL-C, high-density lipoprotein and AIP, atherogenic index of plasma*.

### Association Between AIP and Lipid Levels and 3-Month Prognosis After Stroke

Multivariate logistic analysis revealed that the AIP, as a continuity variable (OR 1.55, 95% CI, 1.35–1.82, *p* = 0.009), was independently associated with poor outcomes at 3 months after the adjustment for age, gender, baseline NIHSS, reperfusion therapy, history of lipid-lowering therapy, history of atrial fibrillation, and history of diabetes mellitus, glucose, TC, and LDL-C. TG (OR 0.59, 95% CI, 0.39–0.91, *p* = 0.017), LDL-C (OR 0.47, 95% CI, 0.32–0.70, *p* = 0.031), and non-HDL-C (OR 1.46, 95% CI, 1.32–1.69, *p* = 0.010) remained prominently associated (Supplementary Table 1). Spline regression showed a dose–response relationship between AIP levels with risk of unfavorable outcomes, as shown in Supplementary Figure 2. It did not reach statistical significance for non-linear association with death and major disability (*p* = 0.217), death (*p* = 0.542), and death or disability (*p* = 0.159).

**Figure 2:**
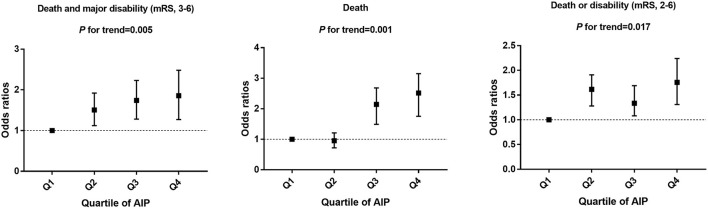
Multivariable adjusted odds ratios for functional outcomes, grouped by AIP quartile in patients.

As shown in Table 2 and Figure 2, compared with the lowest quartile of AIP, ORs (95% CI) with the highest quartile were 1.84 (1.23–2.53) for poor outcomes, 2.64 (1.65–3.23) for disability, 1.73 (1.41–2.32) for death, and 1.82 (1.59–2.17) for 1-U higher mRS score after multivariable adjustment. Every 1-SD increase in AIP level was positively correlated with poor prognosis in AIS patients (*p* = 0.008). Furthermore, high TG (OR 0.39, 95% CI 0.26–0.61 for Q4:Q1, *p* = 0.001) was a protective factor for good outcomes. High non-HDL-C (OR 1.61, 95% CI 1.41–1.89 for Q4:Q1, *p* = 0.021) was closely associated with poor outcomes (Supplementary Table 2).

**Table 2:** ORs (95% CI) for different outcomes associated with AIP after AIS.

**Outcomes**	**AIP**				
	**Quartile 1 < -0.10**	**Quartile 2 −0.10 to 0.08**	**Quartile 3 0.08–0.26**	**Quartile 4 ≥0.26**	***p* trend**
No. of cases	366	366	366	365	
**Primary outcome**					
Death and major disability (mRS, 3–6)					
No. of cases (%)	42 (11.5)	67 (18.3)	70 (19.1)	78 (21.1)	
Multivariable adjusted model^*^	1.00	1.43 (1.11–1.72)	1.62 (1.31–2.12)	1.84 (1.23–2.53)	0.007
**Secondary outcomes**					
**Death**					
No. of cases (%)	7 (1.9)	5 (1.4)	16 (4.4)	20 (5.5)	
Multivariable adjusted model^*^	1.00	0.82 (0.61–1.12)	2.31 (1.45–2.71)	2.64 (1.65–3.23)	0.003
**Death or disability (mRS, 2–6)**					
No. of cases (%)	108 (29.5)	126 (34.4)	109 (29.8)	127 (34.5)	
Multivariable adjusted model^*^	1.00	1.68 (1.23–1.97)	1.34 (1.03–1.72)	1.73 (1.41–2.32)	0.015
Modified Rankin Scale^**^					
0 (no symptoms)	156 (42.6)	144 (39.3)	123 (33.6)	104 (28.8)	
1 (no significant disability despite symptoms)	102 (27.9)	96 (26.2)	134 (36.6)	134 (37.1)	
2 (slight disability)	66 (18.1)	59 (16.1)	39 (10.7)	49 (13.4)	
3 (moderate disability)	19 (5.2)	39 (10.7)	29 (7.9)	34 (8.8)	
4 (moderately severe disability)	15 (4.1)	19 (5.2)	22 (6.0)	20 (5.5)	
5 (severe disability)	1 (0.3)	4 (1.1)	3 (0.8)	4 (1.1)	
6 (dead)	7 (1.9)	5 (1.4)	16 (4.4)	20 (5.5)	
Multivariable adjusted model	1.00	1.64 (1.24–1.82)	1.71 (1.34–1.98)	1.82 (1.59–2.17)	0.008

* *Adjustment for age, gender, and baseline NIHSS; history of lipid-lowering therapy; reperfusion therapy; history of atrial fibrillation; and history of diabetes mellitus, glucose, TC, and LDL-C*.

** *Odds of a 1-U higher modified Rankin Scale score*.

*AIP, atherogenic index of plasma*.

**Figure 3:**
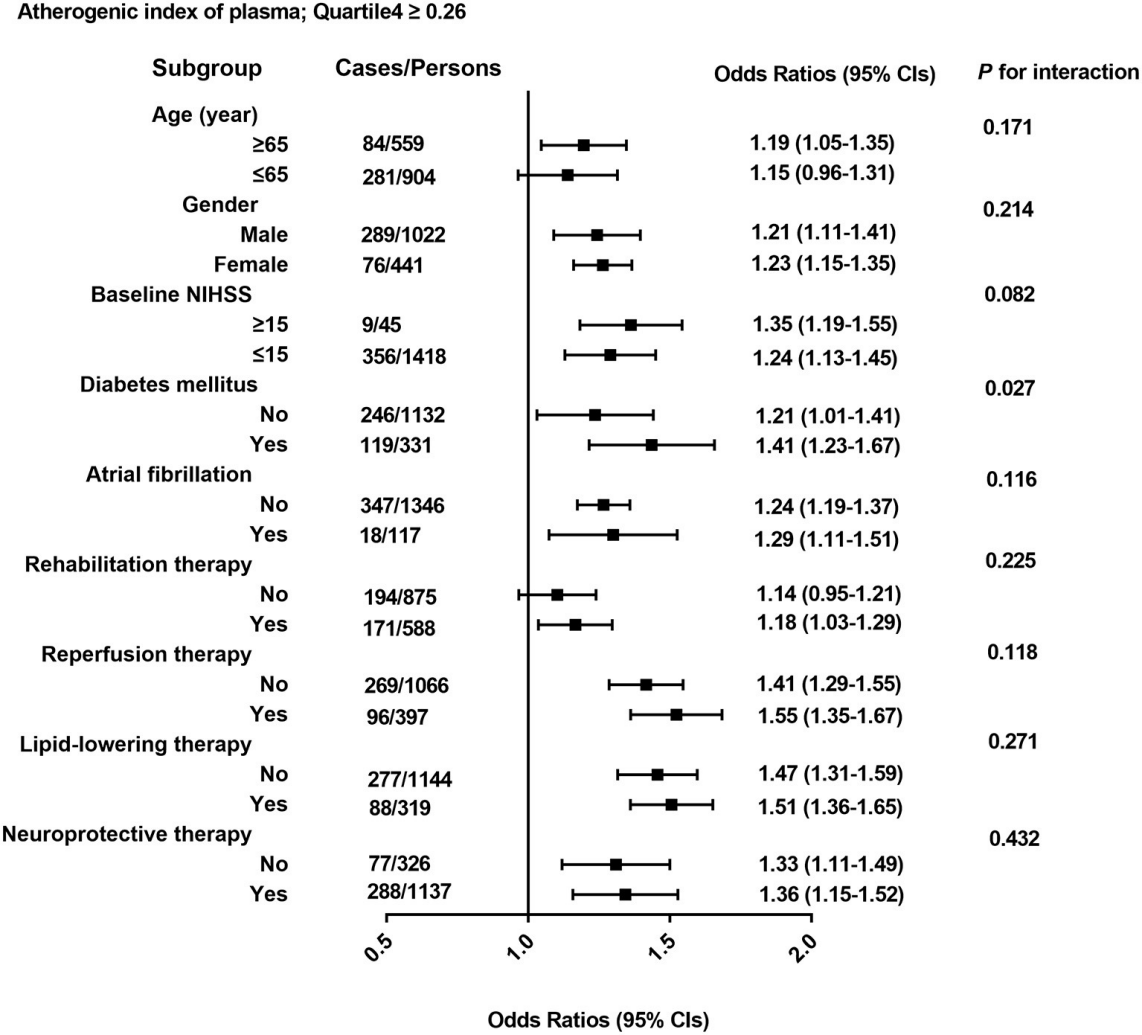
All odds ratios were calculated with AIP. Quartile < -0.10 as the reference groups, with models adjusted for age, gender, and baseline NIHSS; history of lipid-lowering therapy; reperfusion therapy; history of atrial fibrillation; and history of diabetes mellitus, glucose, TC, and LDL-C. Each group adjusted for the other covariates except itself.

As shown in Figure 3, the association of 3-month outcomes of AIS with AIP seemed to be more obvious among patients who were ≥65 years of age (OR 1.19; 95% CI 1.05–1.35), or with higher baseline NIHSS score (OR 1.35; 95% CI 1.19–1.55) or diabetes (OR 1.41; 95% CI 1.23–1.67) or atrial fibrillation (OR 1.29; 95% CI 1.11–1.51) or other therapy methods, but no interaction was observed except for diabetes (*p* for interaction = 0.027).

### Association Between AIP and Functional Outcomes Among Different Stroke Etiology

TOAST subgroup analysis is shown in Table 3. Compared with the lowest quartile of AIP, ORs (95% CI) with the highest quartile were 1.90 (1.53–2.62) for poor outcomes (*p* = 0.002), 2.94 (2.36–3.46) for death (*p* = 0.001), and 1.77 (1.45–2.20) for disability (*p* = 0.007) among stroke patients of LAA subtype after multivariable adjustment. Other stroke types had no statistical significance.

**Table 3:** Adjusted odds ratios of outcomes according to AIP in stroke subtypes.

**Stroke subtypes**	**AIP**				
	**Quartile 1 < -0.10**	**Quartile 2 −0.10 to 0.08**	**Quartile 3 0.08–0.26**	**Quartile 4 ≥0.26**	***p^#^* trend**
No. of cases	366	366	366	365	
**Large artery atherosclerosis**					
Model 1^*^	1	1.29 (0.77–2.18)	1.58 (1.32–1.87)	1.90 (1.53–2.62)	0.002
Model 2^**^	1	1.13 (1.03–1.58)	2.43 (2.15–3.20)	2.94 (2.36–3.46)	0.001
Model 3^***^	1	1.45 (1.26–1.78)	1.70 (1.43–2.16)	1.77 (1.45–2.20)	0.007
**Cardioembolic**					
Model 1^*^	1	0.12 (0.01–1.03)	0.18 (0.02–1.65)	0.21 (0.02–1.87)	0.115
Model 2^**^	1	0.17 (0.02–1.74)	1.51 (0.33–6.89)	0.28 (0.03–2.79)	0.157
Model 3^***^	1	0.55 (0.18–3.63)	4.11 (1.02–16.4)	0.93 (0.21–4.21)	0.114
**Small-artery occlusion**					
Model 1^*^	1	0.77 (0.34–1.76)	1.05 (0.48–2.26)	0.50 (0.19–1.31)	0.439
Model 2^**^	1	0.91 (0.12–6.67)	1.51 (0.24–9.23)	0.58 (0.05–6.48)	0.859
Model 3^***^	1	0.51 (0.25–1.03)	0.85 (0.44–1.63)	0.74 (0.37–1.48)	0.311
**Other or unknown cause**					
Model 1^*^	1	1.25 (0.51–3.06)	1.27 (0.49–3.25)	0.75 (0.29–1.92)	0.672
Model 2^**^	1	1.91 (0.44–8.31)	1.86 (0.39–8.66)	0.31 (0.03–3.02)	0.343
Model 3^***^	1	1.62 (0.78–3.33)	1.41 (0.65–3.03)	0.81 (0.38–1.71)	0.241

# *Adjustment for age, gender, and baseline NIHSS; history of lipid-lowering therapy; reperfusion therapy; history of atrial fibrillation; and history of diabetes mellitus, glucose, TC, and LDL-C*.

* *Death and major disability (mRS, 3–6)*.

** *Death (mRS = 6)*.

*** *Death or disability (mRS, 2–6)*.

*AIP, atherogenic index of plasma*.

### Predictive Values of AIP and Lipid Levels for Outcomes at 3 Months After Stroke

**Figure 4:**
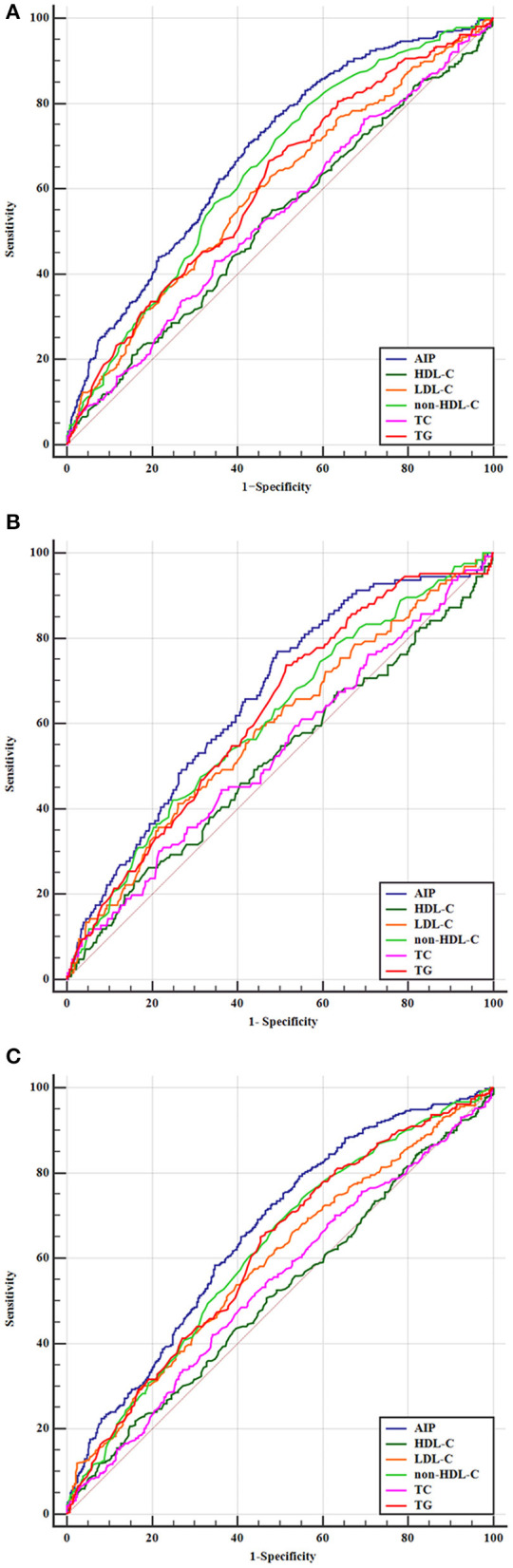
Predictive values of TC, TG, LDL-C, HDL-C, non-HDL-C, and AIP for the outcomes. Receiver operating characteristic curves for outcomes. **(A)** Areas under the curves for poor outcomes: 0.611 for TG, 0.537 for TC, 0.595 for LDL-C, 0.525 for HDL-C, 0.645 for non-HDL-C, and 0.685 for AIP. **(B)** Areas under the curves for death: 0.624 for TG, 0.536 for TC, 0.588 for LDL-C, 0.516 for HDL-C, 0.608 for non-HDL-C, and 0.663 for AIP. **(C)** Areas under the curves for disability: 0.613 for TG, 0.532 for TC, 0.589 for LDL-C, 0.512 for HDL-C, 0.617 for non-HDL-C, and 0.661 for AIP.

To further evaluate the predictive values of TC, TG, LDL-C, HDL-C, non-HDL-C, and AIP for AIS, the receiver operator characteristic curves and AUCs regarding poor outcomes, death, and disability were created and are depicted in Figure 4. The best discriminating variable was the AIP, which showed the highest AUC value than other lipid variables (*p* < 0.001) in poor outcomes (AUC = 0.685, 95% CI 0.652–0.717), death (AUC = 0.663, 95% CI 0.629–0.695), and disability (AUC = 0.661, 95% CI 0.627–0.695). The best predictive cutoff value was 0.112 in poor outcomes (sensitivity 70.8% and specificity 59.2%), 0.109 in death (sensitivity 72.7% and specificity 64.6%), and 0.116 in disability (sensitivity 77.3% and specificity 61.5%). The positive/negative likelihood ratios and diagnostic odds ratio for each predictive marker are shown in Supplementary Table 3.

The comparative analysis of the predictive ability between AIP and NIHSS is shown in Supplementary Figure 3. Compared with AIP, the predictive AUC value of the NIHSS score was 0.724, 95% CI 0.702–0.764, in poor outcomes. However, although NIHSS scores showed slightly higher predictive values compared to AIP, there was no statistical significance (*p* = 0.064). Combining the two models showed higher predictive values. The same results were also shown in the two other end points.

## Discussion

In the present study, our results showed that high AIP value was associated with risk of poor prognosis within 3 months of AIS. Furthermore, the AIP represented a clinically available prognostic predictor and had a better predictive value than other lipid levels in the current analysis. Compared with other types of stroke, high AIP level in patients with LAA was closely associated with poor outcomes.

As shown in various pathologic conditions, studies have shown that AS was the most common cause of AIS, and dyslipidemia was the most important risk factor of AS (3, 4). Many blood lipid parameters have been used to evaluate the risk of stroke outcomes, such as TG, TC, LDL-C, HDL-C, non-HDL-C, and other parameters (5–7). However, most studies have shown that the traditional single index of blood lipid as the evaluation of AS and cerebrovascular diseases was still debated and not of high predictive value (19). Recently, the AIP, which was calculated by the log10 of TG to HDL-C ratio had emerged as a novel marker of dyslipidemia and was more stable than other lipid counts alone (13). Furthermore, compared with the unconverted variables, the logarithmic conversion value of TG/HDL-C to calculate AIP, which was used to describe the clinical prediction effect, could better satisfy the statistical model assumed as ecological distribution (20). Several studies have identified AIP as a prognostic biomarker of cardiovascular disease. Wu et al. conducted a case–control study involving 696 individuals (348 patients and 348 controls). Results showed that AIP was identified as an independent risk factor for cardiovascular disease after adjusting for traditional risk factors (21). A study by Garg et al. enrolled 267 patients who were referred to carotid artery stenosis; the findings demonstrated that the AIP was the only lipid parameter independently associated with symptomatic carotid artery stenosis (22). Another study of 1,131 Chinese patients who underwent selective coronary angiography found that AIP as a new marker appeared to be an independent predictor of CVD severity (23). Won et al. included 1,488 patients who underwent serial coronary computed tomography angiography with a median inter-scan period of 3.4 years and revealed that AIP was an independent predictor of rapid plaque progression outperformed traditional risk factors (24). However, few studies have examined the relationship between AIP and functional outcomes in AIS. Therefore, larger sample size studies and prospective cohort studies are needed to explore this relationship.

In our study, AIP was used as a continuous variable and a categorical variable, respectively. Spline regression showed a dose–response relationship between AIP levels with risk of poor outcomes. After controlling for other confounding factors, we used restricted cubic splines with five knots at the 5th, 35th, 50th, 65th, and 95th centiles to flexibly model the association of AIP with poor outcomes. The results showed a J-shaped curve. Furthermore, as categorical variable, previous studies have shown that dividing patients into four groups may better reflect the efficacy of AIP in different groups (25). Our study showed that a high AIP value was associated with risk of poor prognosis within 3 months of AIS and had a higher predictive value than other lipid factors. Several speculations may explain this phenomenon. In the process of stroke occurrence, studies have shown that different sizes of lipid particles have different effects on stroke, especially small dense low-density lipoprotein (sdLDL), which was the most closely related to AIS and was an independent risk factor (26). A large number of sdLDL could easily pass through the vascular endothelium and combined with the glycoprotein on the arterial wall to form lipid deposition (27). Furthermore, apoB100 of sdLDL was difficult to combine with plasma LDL receptors, which greatly reduces the clearance rate (28). On the other hand, sdLDL was easily oxidized to oxidized LDL-C, which caused the aggregation of adhesion molecules and chemokines and induced the transformation of monocytes to macrophages (29). Macrophage phagocytes oxidized LDL-C to produce foam cells. Foam cells could fuse and burst, releasing large amounts of cholesterol that form the core of atherosclerotic plaque. This process could also aggravate AS and affect the outcomes of stroke (30–32). Dobiasova et al. found that the AIP value was inversely proportional to the diameter of oxidized LDL particles and indirectly showed sdLDL particle size, which could be used as an indirect method to reflect LDL particle size instead (33). We speculated that the calculation of the AIP value could more accurately evaluate the tendency of blood lipid-induced stroke.

Our results also showed that compared with other conventional lipid markers, AIP demonstrated a higher predictive value. However, this predictive value is not superior to traditional NIHSS. With further research, we found that combining AIP and NIHSS scores into one variable can significantly improve the predictive value of the model. In order to predict the prognosis of stroke better, more composite indicators may be needed in the future.

In the study, subgroup analysis showed that high levels of AIP were associated with stroke subtypes, especially in the LAA stroke subtype. Some studies have verified that AS plays a important role in LAA stroke (34). AIP may reflect subtle metabolic interactions throughout the lipoprotein complex (11). The etiological mechanism of different stroke subtypes is different, which is of great significance for treatment and prognosis evaluation (35). Up to now, few studies havec investigated the relationship between AIP and the outcomes of different stroke subtypes. We hypothesize that AIP may not have the equal contribution on the prognosis of patients with LAA and other stroke subtypes. We also found that the association of 3-month outcomes of AIS with AIP seemed to be more obvious among patients with diabetes. It was plausible to suppose that these traditional cardiovascular risk factors may amplify the detrimental effect of high level of AIP on poor prognosis of stroke. Previous studies showed that LAA is the most common stroke subtype in patients with AIS with type 2 diabetes (36, 37). We supposed that there may be the following reasons. The high glucose level in diabetics accelerates the formation of protein glycosylation end products, causing them to accumulate in tissues, causing proliferation of smooth muscle cells and thickening of blood vessel walls. Lipid deposits in the intima of blood vessels form fat stripes and cause intima thickening. Then, there was local aggregation of complex sugars and lipids, fibrous tissue hyperplasia, and finally plaque formation leading to lumen stenosis or even occlusion.

Several limitations remained in this study. First, the study was a single center and cannot avoid some selection bias. Second, only baseline AIP was available in our study. Dynamic changes of AIP during the follow-up were not available. However, the Framingham Offspring cohort showed that serum lipid parameter concentrations were usually stable over the 30-year life course (38). Third, follow-up was only by telephone in this study, and end-point assessment could not be validated by hospital records in all individuals. Finally, some imaging data were not provided for readers to evaluate the degree of AS among this cohort. Further large-scaled cohort studies in other populations are needed to verify the generalizability of our findings.

## Conclusion

In summary, our findings suggested that high AIP value was associated with death and poor outcomes in patients with AIS, especially the stroke subtype of LAA. In medical practice, the AIP level may be easily applied to distinguish poor outcomes and provided a novel target for neuroprotection in AIS patients.

## Data Availability Statement

The raw data supporting the conclusions of this article will be made available by the authors, without undue reservation.

## Ethics Statement

Written informed consent was obtained from the individual(s) for the publication of any potentially identifiable images or data included in this article.

## Author Contributions

YX and BS: research design. YG and HL: writing. SL, LZ, and HF: data analysis and arrangement. LP and KL: English editing help. KZ, CZ, and JZ: data collection. SS and JW: tables and figures. All authors contributed to the article and approved the submitted version.

## Funding

This research has been supported by the China National Key R&D Program during the 13th Five-year Plan Period (Grant No. 2018YFC1311303) and Major Science and Technology Projects of Henan Province in 2020 (Grant No. 201300310300).

## Conflict of Interest

The authors declare that the research was conducted in the absence of any commercial or financial relationships that could be construed as a potential conflict of interest.

## Publisher's Note

All claims expressed in this article are solely those of the authors and do not necessarily represent those of their affiliated organizations, or those of the publisher, the editors and the reviewers. Any product that may be evaluated in this article, or claim that may be made by its manufacturer, is not guaranteed or endorsed by the publisher.

## Supplementary Material

Supplementary Figure 1 Comparison of TC, TG, LDL-C, HDL-C, non-HDL-C, AIP quartile results in AIS patients.

Supplementary Figure 2 Association of AIP level to the risk of poor outcomes based on spline regression model.

Supplementary Figure 3 Predictive values of AIP and NIHSS for the outcomes. Receiver operating characteristic curves for outcomes.

Supplementary Table 1 Multivariate logistic regression analysis predicting prognosis after AIS, as continuity variables.

Supplementary Table 2 Adjusted odds ratios for prognosis according to routine blood lipid variables.

Supplementary Table 3 The positive/negative likelihood ratios and diagnostic odds ratio for each predictive marker.
