# Supplementary figures and images for: Atherogenic Index of Plasma Predicts Outcomes in Acute Ischemic Stroke
Source: Front Neurol. 2021 Oct 11;12:741754. doi: 10.3389/fneur.2021.741754 (PMC8542679; doi:10.3389/fneur.2021.741754)

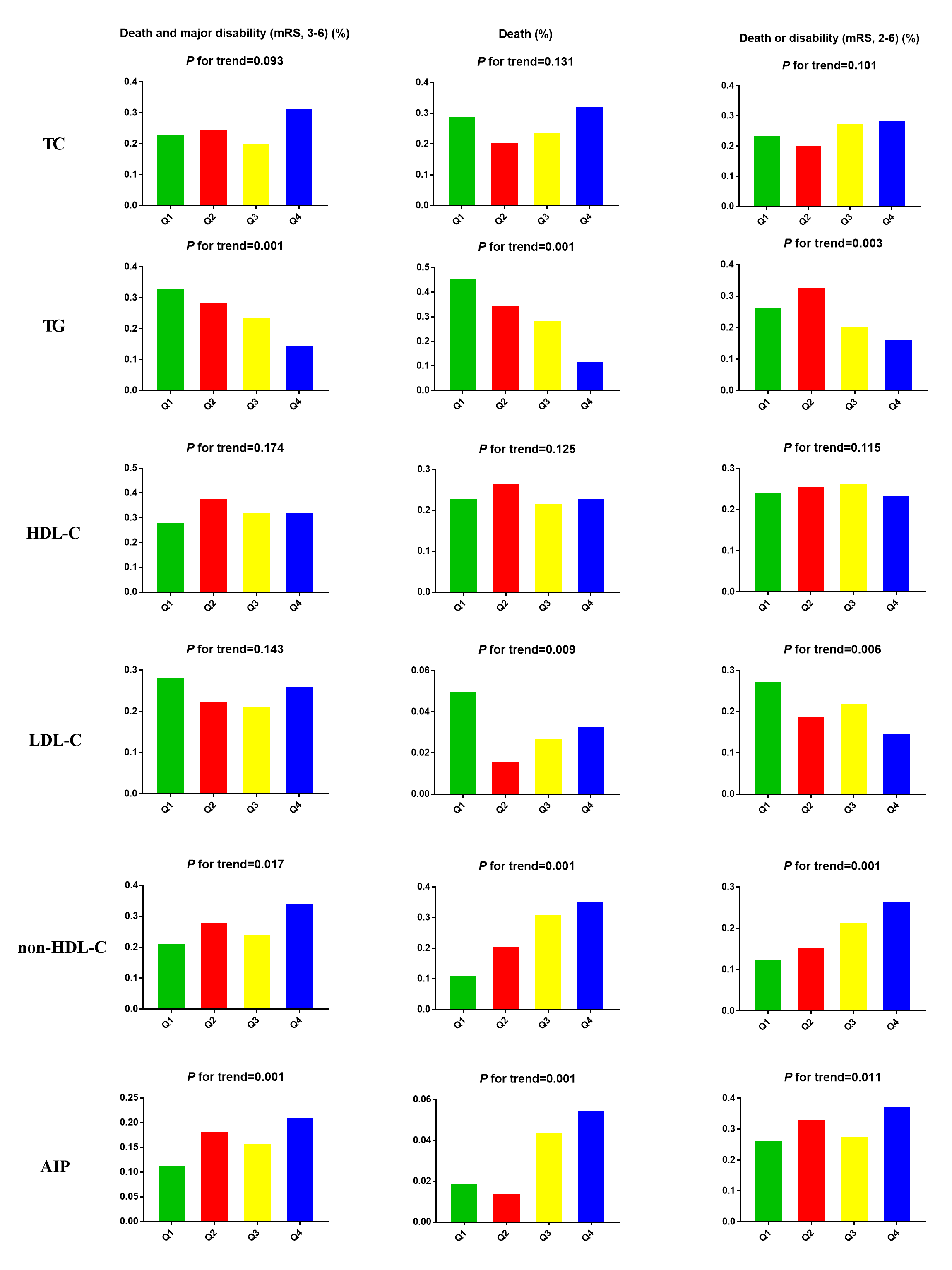

Supplement: Supplementary Figure 1 — Comparison of TC, TG, LDL-C, HDL-C, non-HDL-C, AIP quartile results in AIS patients. [file Image_1.TIF]

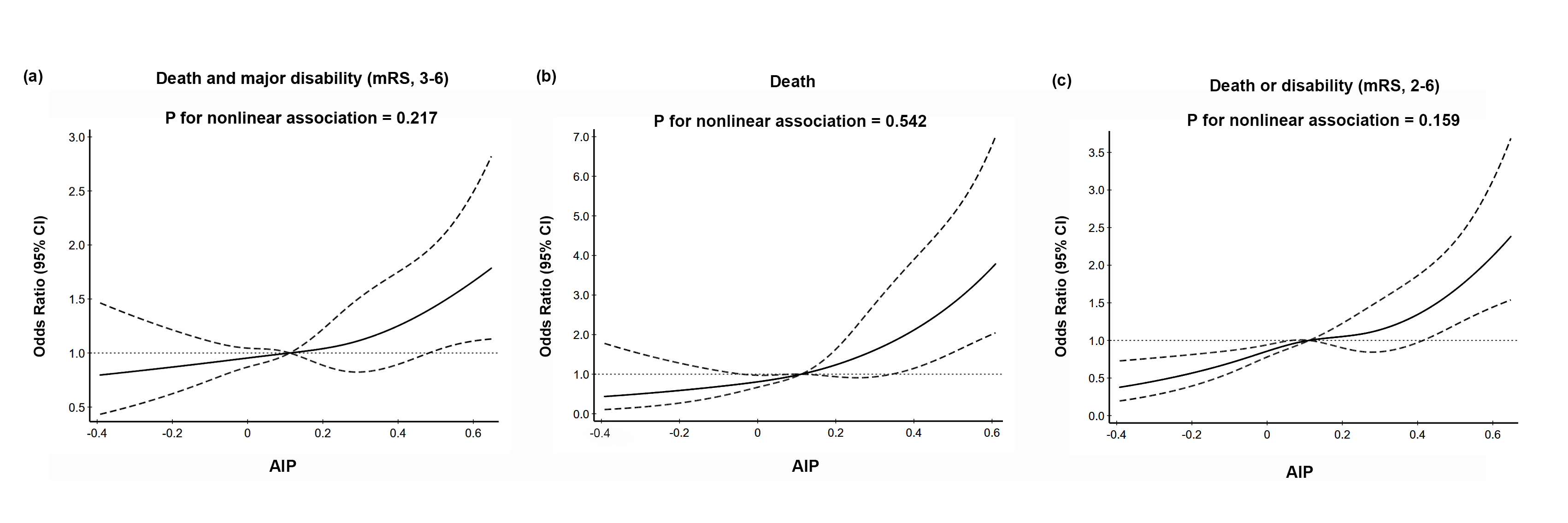

Supplement: Supplementary Figure 2 — Association of AIP level to the risk of poor outcomes based on spline regression model. [file Image_2.TIF]

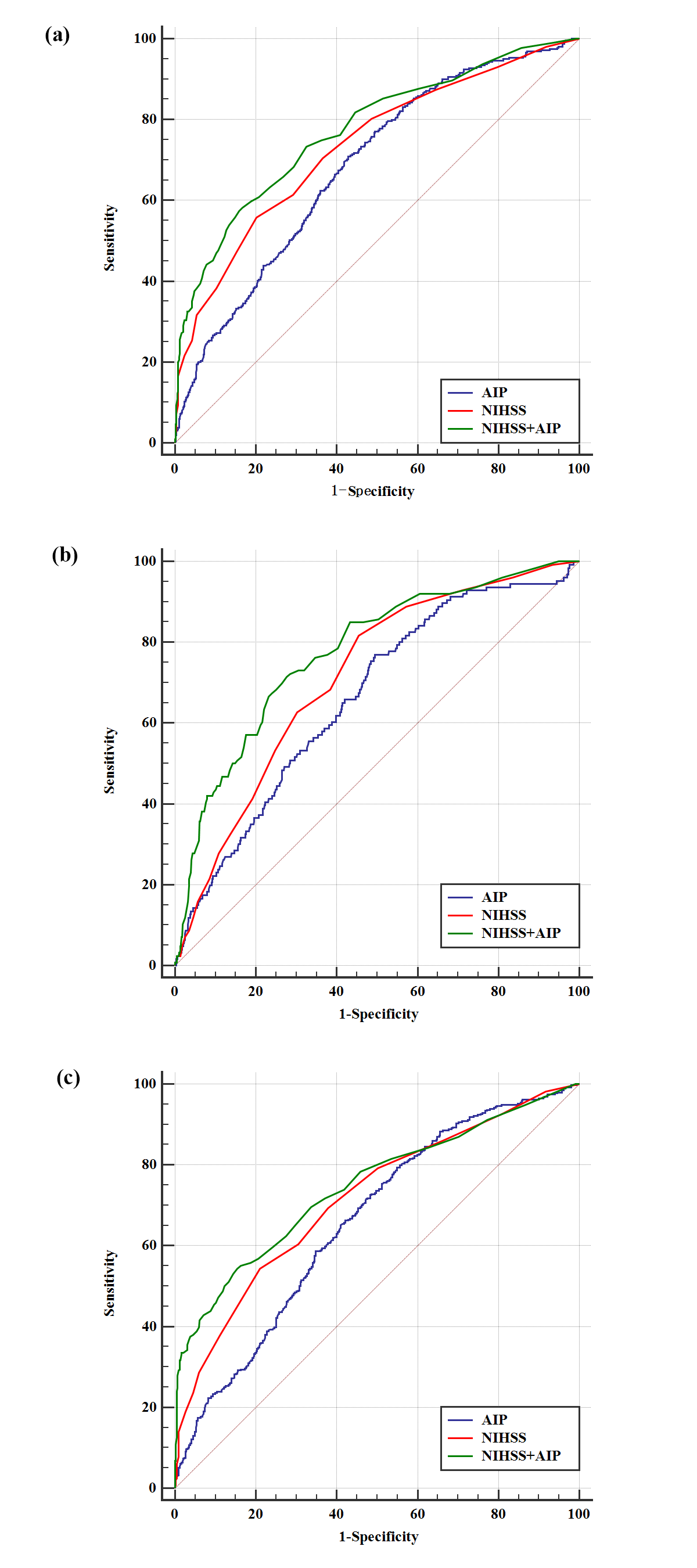

Supplement: Supplementary Figure 3 — Predictive values of AIP and NIHSS for the outcomes. Receiver operating characteristic curves for outcomes. [file Image_3.TIF]
