# Supplementary material for: Atherogenic Index of Plasma Predicts Outcomes in Acute Ischemic Stroke
Source: Front Neurol. 2021 Oct 11;12:741754. doi: 10.3389/fneur.2021.741754 (PMC8542679; doi:10.3389/fneur.2021.741754)
Supplement: Supplementary Table 1 — Multivariate logistic regression analysis predicting prognosis after AIS, as continuity variables. [file Data_Sheet_1.docx]

**Supplemental table 1.Multivariate logistic regression analysis predictingprognosis after AIS, as continuity variables.**

| Model | Independent variable | Multivariate logistic regression analysis* | | |
| --- | --- | --- | --- | --- |
|  |  | OR | 95% CI | P Value |
| Model 1  (with Routine lipid variables) | Age, y | 1.03 | 1.02-1.05 | 0.001 |
|  | Male | 1.05 | 0.76-1.47 | 0.746 |
|  | Baseline NIHSS | 1.26 | 1.22-1.31 | 0.001 |
|  | Reperfusion therapy | 1.38 | 1.11-1.85 | 0.032 |
|  | History of lipid-lowering therapy | 1.78 | 0.71-1.47 | 0.119 |
|  | History of atrial fibrillation | 1.88 | 1.03-3.43 | 0.039 |
|  | History of diabetes mellitus | 1.53 | 1.08-2.15 | 0.016 |
|  | Glucose (mmol/L) | 1.07 | 1.01-1.13 | 0.017 |
|  | TC (mmol/L) | 1.05 | 0.94-1.18 | 0.395 |
|  | TG (mmol/L) | 0.59 | 0.39-0.91 | 0.017 |
|  | LDL-C (mmol/L) | 0.47 | 0.32-0.70 | 0.031 |
|  | HDL-C (mmol/L) | 0.66 | 0.23-1.12 | 0.098 |
| Model 2 (with non-HDL-C) | Age, y | 1.04 | 1.01-1.04 | 0.001 |
|  | Male | 1.01 | 0.72-1.41 | 0.957 |
|  | Baseline NIHSS | 1.26 | 1.12-1.33 | 0.001 |
|  | Reperfusion therapy | 1.32 | 1.15-1.88 | 0.056 |
|  | History of lipid-lowering therapy | 1.94 | 0.54-1.56 | 0.126 |
|  | History of atrial fibrillation | 1.43 | 0.87-2.63 | 0.099 |
|  | History of diabetes mellitus | 1.55 | 1.09-2.09 | 0.014 |
|  | Glucose (mmol/L) | 1.12 | 1.04-1.15 | 0.011 |
|  | TG (mmol/L) | 0.64 | 0.43-0.94 | 0.023 |
|  | LDL-C (mmol/L) | 0.83 | 0.72-0.96 | 0.013 |
|  | non-HDL-C (mmol/L) | 1.46 | 1.32-1.69 | 0.010 |
| Model 3 (with AIP) | Age, y | 1.02 | 1.02-1.04 | 0.001 |
|  | Male | 1.07 | 0.75-1.55 | 0.722 |
|  | Baseline NIHSS | 1.27 | 1.22-1.31 | 0.001 |
|  | Reperfusion therapy | 1.31 | 1.16-1.54 | 0.027 |
|  | History of lipid-lowering therapy | 1.87 | 0.74-1.32 | 0.264 |
|  | History of atrial fibrillation | 1.51 | 0.91-2.16 | 0.071 |
|  | History of diabetes mellitus | 1.18 | 1.03-1.35 | 0.016 |
|  | Glucose (mmol/L) | 1.09 | 1.02-1.27 | 0.021 |
|  | TC (mmol/L) | 1.22 | 0.95-1.57 | 0.104 |
|  | LDL-C (mmol/L) | 0.81 | 0.70-0.95 | 0.019 |
|  | AIP | 1.55 | 1.35-1.82 | 0.009 |

NIHSS, national institutes of health stroke scale; TC, total cholesterol; TG, triglyceride; LDL-C, low-density lipoprotein; HDL-C, high-density lipoprotein and AIP,atherogenic index of plasma.

| Stroke subtypes | Lipid measures | | | | |
| --- | --- | --- | --- | --- | --- |
|  | Quartile1 | Quartile2 | Quartile3 | Quartile4 | *P*Trend^a^ |
| TC (mmol/L) | <3.46 | 3.46-4.11 | 4.11-4.78 | ≥4.78 |  |
| Model 1^*^ | 1 | 1.08 (0.73-1.60) | 0.86 (0.57-1.29) | 1.44 (0.99-2.10) | 0.155 |
| Model 2^**^ | 1 | 0.49 (0.19-1.23) | 0.71 (0.31-1.61) | 1.22 (0.59-2.52) | 0.187 |
| Model 3^***^ | 1 | 0.81 (0.59-1.12) | 1.24 (0.92-1.71) | 1.33 (0.97-1.81) | 0.110 |
| TG (mmol/L) | <0.92 | 0.92-1.28 | 1.28-1.78 | ≥1.78 |  |
| Model 1^*^ | 1 | 0.85 (0.59-1.23) | 0.68 (0.47-0.99) | 0.39 (0.26-0.61) | 0.001 |
| Model 2^**^ | 1 | 0.68 (0.53-1.04) | 0.51 (0.22-1.23) | 0.15 (0.07-0.41) | 0.001 |
| Model 3^***^ | 1 | 1.31 (0.96-1.77) | 0.87 (0.64-1.19) | 0.67 (0.49-0.93) | 0.007 |
| HDL-C (mmol/L) | <0.91 | 0.91-1.06 | 1.06-1.27 | ≥1.27 |  |
| Model 1^*^ | 1 | 1.54 (1.03-2.31) | 1.41 (0.94-2.11) | 1.41 (0.92-2.17) | 0.177 |
| Model 2^**^ | 1 | 1.31 (0.95-2.67) | 0.99 (0.42-2.35) | 1.05 (0.56-2.12) | 0.093 |
| Model 3^***^ | 1 | 1.04 (0.76-1.43) | 1.13 (0.83-1.56) | 1.18 (0.86-1.61) | 0.216 |
| LDL-C (mmol/L) | <2.01 | 2.01-2.54 | 2.54-3.13 | ≥3.13 |  |
| Model 1^*^ | 1 | 0.76 (0.51-1.12) | 0.71 (0.48-1.05) | 0.89 (0.68-1.14) | 0.085 |
| Model 2^**^ | 1 | 0.29 (0.15-0.72) | 0.49 (0.23-1.05) | 0.59 (0.28-1.21) | 0.039 |
| Model 3^***^ | 1 | 0.71 (0.52-0.98) | 0.96 (0.73-1.35) | 0.69 (0.38-0.93) | 0.015 |
| non-HDL-C (mmol/L) | <2.41 | 2.41-3.01 | 3.01-3.63 | ≥3.63 | *P*Trend^b^ |
| Model 1^*^ | 1 | 1.59 (1.41-1.87) | 1.71 (1.48-2.02) | 1.61 (1.41-1.89) | 0.021 |
| Model 2^**^ | 1 | 1.29 (1.11-1.59) | 1.51 (1.23-1.87) | 1.90 (1.45-2.79) | 0.016 |
| Model 3^***^ | 1 | 1.46 (1.26-1.62) | 1.55 (1.38-1.89) | 1.65 (1.48-1.89) | 0.012 |
| LDL-C/HDL-C | <1.81 | 1.81-2.39 | 2.39-3.07 | ≥3.07 | *P*Trend^c^ |
| Model 1^*^ | 1 | 0.77 (0.51-1.15) | 1.20 (0.82-1.75) | 1.06 (0.72-1.55) | 0.178 |
| Model 2^**^ | 1 | 0.48 (0.22-1.09) | 0.64 (0.22-1.09) | 0.65 (0.31-1.38) | 0.208 |
| Model 3^***^ | 1 | 1.04 (0.76-1.42) | 1.12 (0.82-1.53) | 1.13 (0.83-1.55) | 0.841 |

**Supplemental table 2.Adjusted odds ratios for prognosis according to routine blood lipid variables.**

TC, TG, HDL-C andLDL-C; *P*^b^,adjustment for*P*^a^but not include TC and HDL-C;*P*^c^,adjustment for*P*^a^but not include HDL-C and LDL-C;

**^*^**Death and major disability (mRS, 3–6);**^**^**Death (mRS=6); ^***^Death or disability (mRS, 2-6)

TC, total cholesterol; TG, triglyceride;HDL-C, high-density lipoprotein;LDL-C, low-density lipoprotein and AIP,atherogenic index of plasma.

| Variable | Cut-off value | Sensitivity,% | Specificity,% | PLR | NLR | DOR |
| --- | --- | --- | --- | --- | --- | --- |
| Death and major disability (mRS, 3–6) |  | | | | |  |
| AIP | 0.11 | 70.8 | 59.2 | 1.74 | 0.49 | 3.52 |
| HDL-C | 2.95 | 53.3 | 53.9 | 1.16 | 0.87 | 1.33 |
| LDL-C | 2.44 | 59.2 | 56.5 | 1.36 | 0.72 | 1.88 |
| Non-HDL-C | 0.93 | 79.6 | 43.1 | 1.39 | 0.47 | 2.96 |
| TC | 4.44 | 42.9 | 65.2 | 1.23 | 0.88 | 1.41 |
| TG | 1.21 | 66.7 | 52.6 | 1.41 | 0.63 | 2.22 |
| Death |  | | | | | |
| AIP | 0.11 | 72.7 | 64.6 | 2.05 | 0.42 | 4.86 |
| HDL-C | 2.11 | 43.1 | 73.1 | 1.61 | 0.78 | 2.06 |
| LDL-C | 2.01 | 41.3 | 73.8 | 1.58 | 0.79 | 1.98 |
| Non-HDL-C | 1.23 | 42.1 | 75.2 | 1.70 | 0.77 | 2.21 |
| TC | 4.92 | 34.5 | 78.4 | 1.60 | 0.84 | 1.91 |
| TG | 1.21 | 73.8 | 48.5 | 1.43 | 0.54 | 2.65 |
| Death or disability (mRS, 2-6) |  | | | | | |
| AIP | 0.12 | 77.3 | 61.5 | 2.01 | 0.37 | 5.44 |
| HDL-C | 2.17 | 33.5 | 79.2 | 1.61 | 0.84 | 1.92 |
| LDL-C | 2.38 | 53.8 | 60.2 | 1.35 | 0.77 | 1.76 |
| Non-HDL-C | 0.94 | 74.1 | 44.5 | 1.34 | 0.58 | 2.29 |
| TC | 4.44 | 42.1 | 65.9 | 1.23 | 0.88 | 1.41 |
| TG | 1.21 | 65.1 | 54.5 | 1.44 | 0.64 | 2.23 |

**Supplemental table 3.The positive/negative likelihood ratios and diagnostic odds ratio for each predictive marker.**

PLR, positive likelihood ratio; NLR, negative likelihood ratio; DOR, diagnostic odds ratio
